# Supplementary figures and images for: Multi-epitope immunocapture of huntingtin reveals striatum-selective molecular signatures
Source: Mol Syst Biol. 2025 Apr 1;21(5):492–522. doi: 10.1038/s44320-025-00096-3 (PMC12048488; doi:10.1038/s44320-025-00096-3)

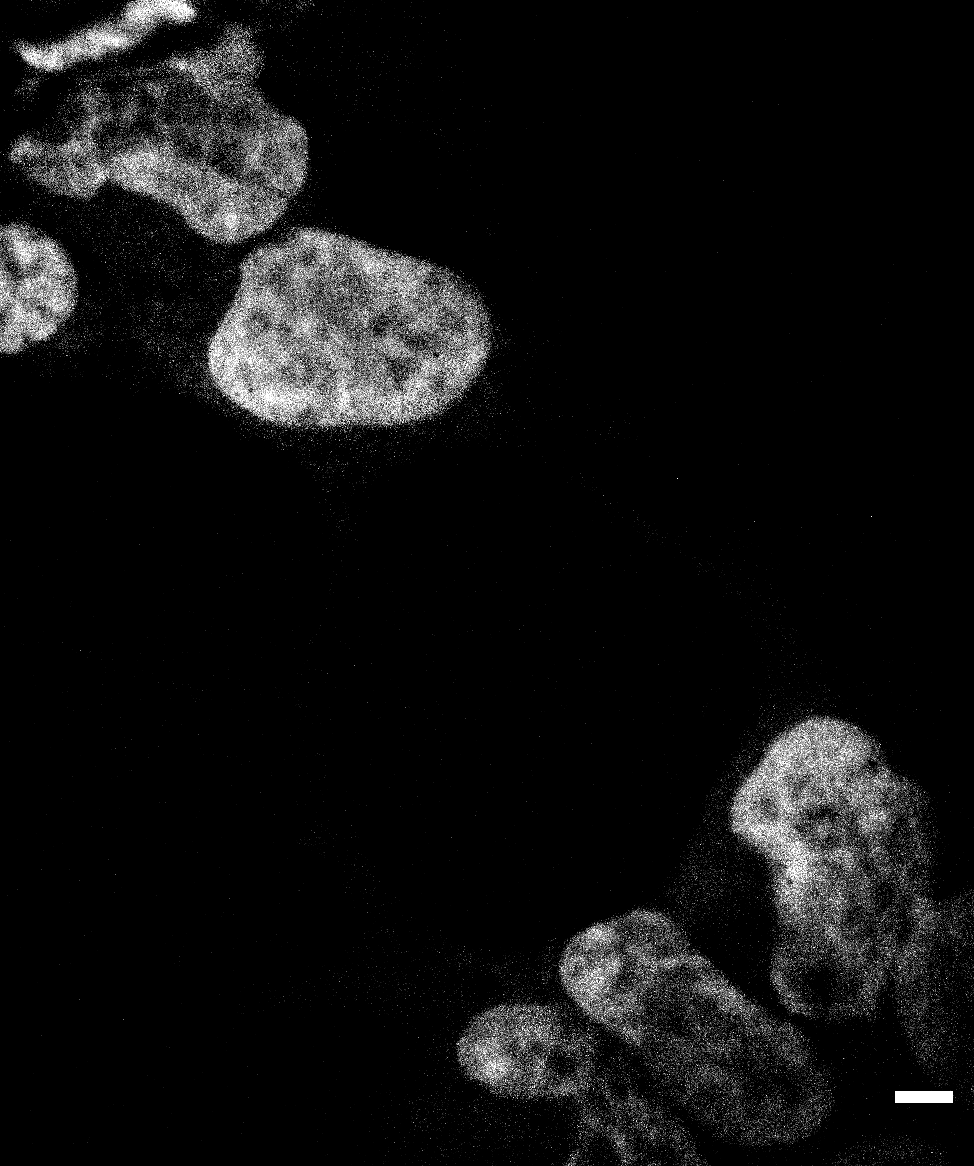

Supplement: Supplementary file 8 — Source data Fig. 2 [file 44320_2025_96_MOESM8_ESM.zip › SD_Images_Fig2K/MAX_4E10_DAPI.png]

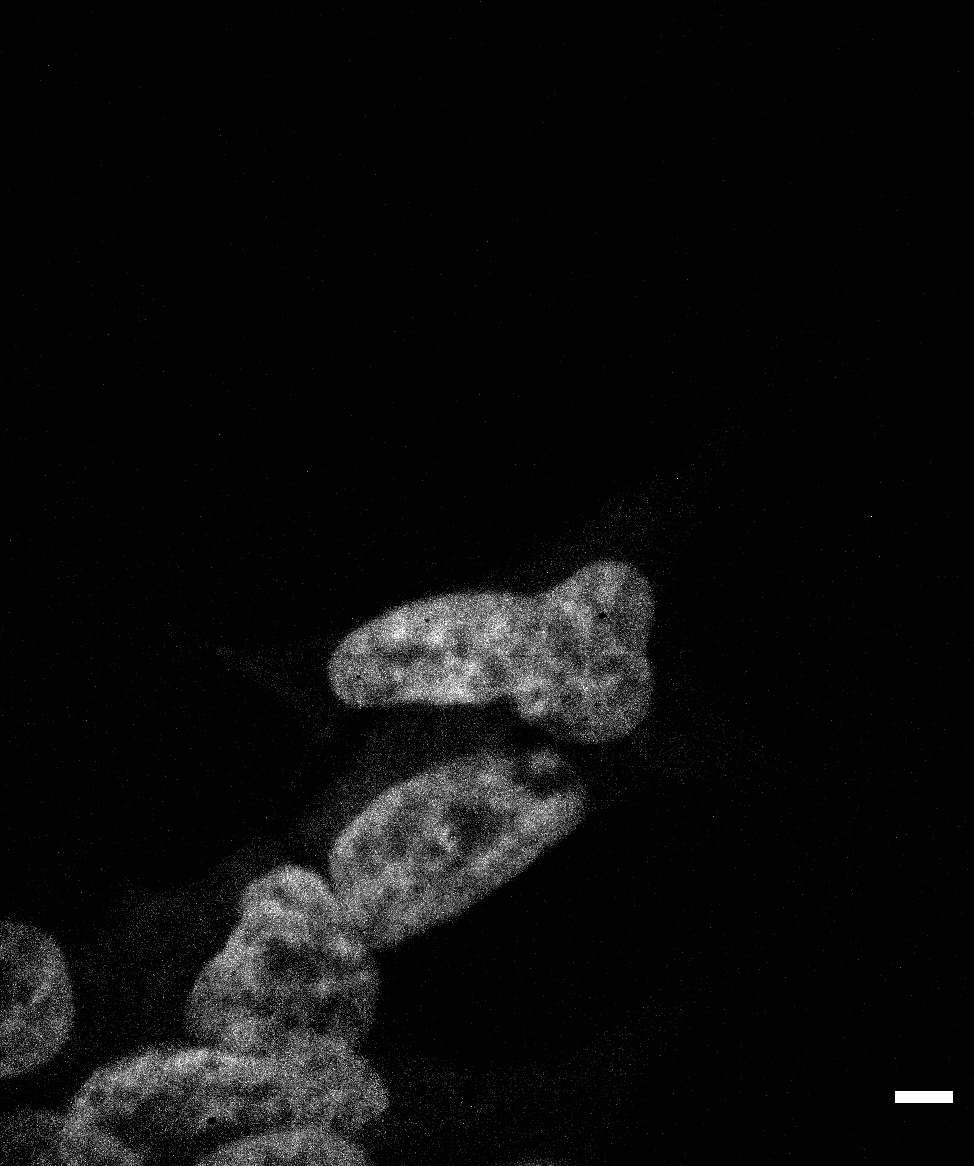

Supplement: Supplementary file 8 — Source data Fig. 2 [file 44320_2025_96_MOESM8_ESM.zip › SD_Images_Fig2K/MAX_2B7_DAPI.png]

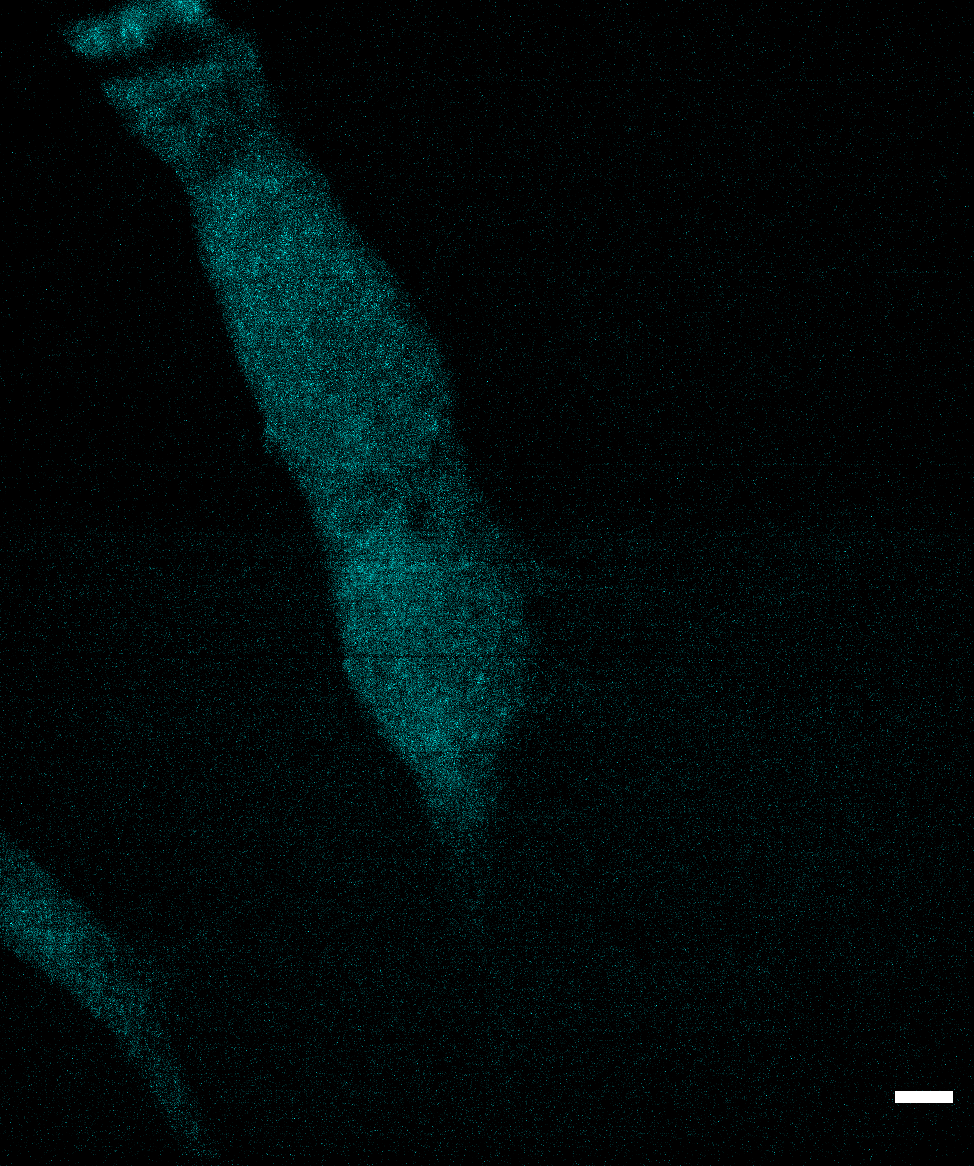

Supplement: Supplementary file 8 — Source data Fig. 2 [file 44320_2025_96_MOESM8_ESM.zip › SD_Images_Fig2K/MAX_3E10_HTT.png]

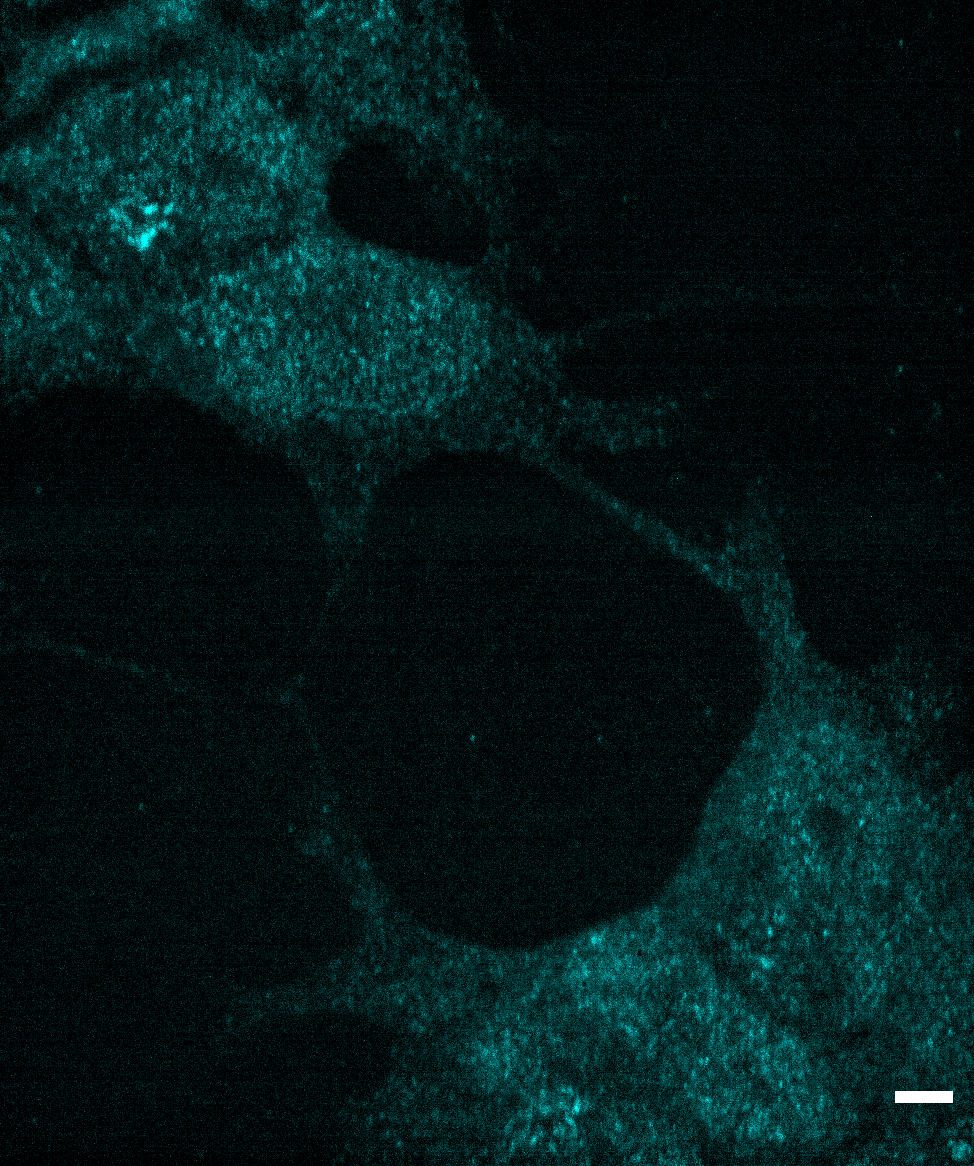

Supplement: Supplementary file 8 — Source data Fig. 2 [file 44320_2025_96_MOESM8_ESM.zip › SD_Images_Fig2K/MAX_4E10_HTT.png]

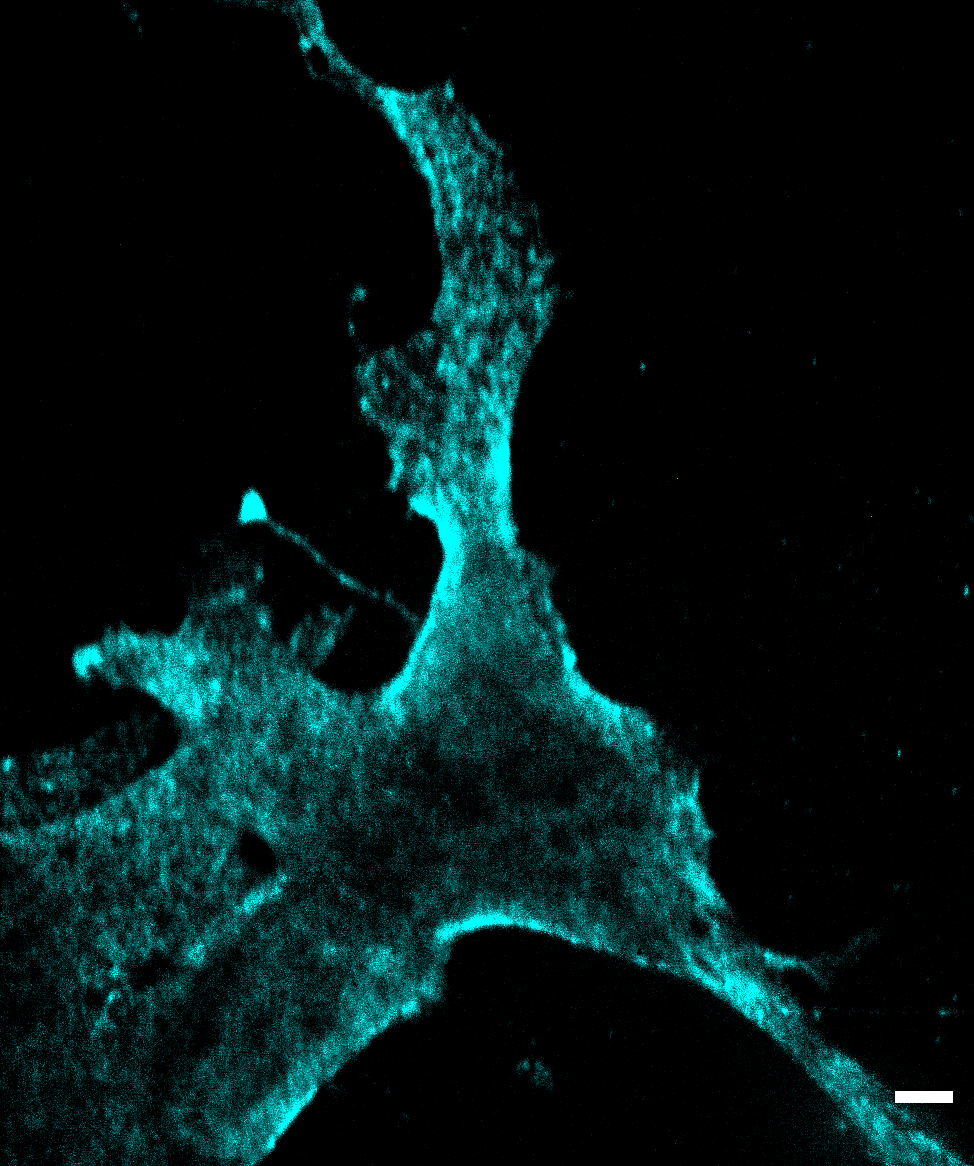

Supplement: Supplementary file 8 — Source data Fig. 2 [file 44320_2025_96_MOESM8_ESM.zip › SD_Images_Fig2K/MAX_4C9_HTT.png]

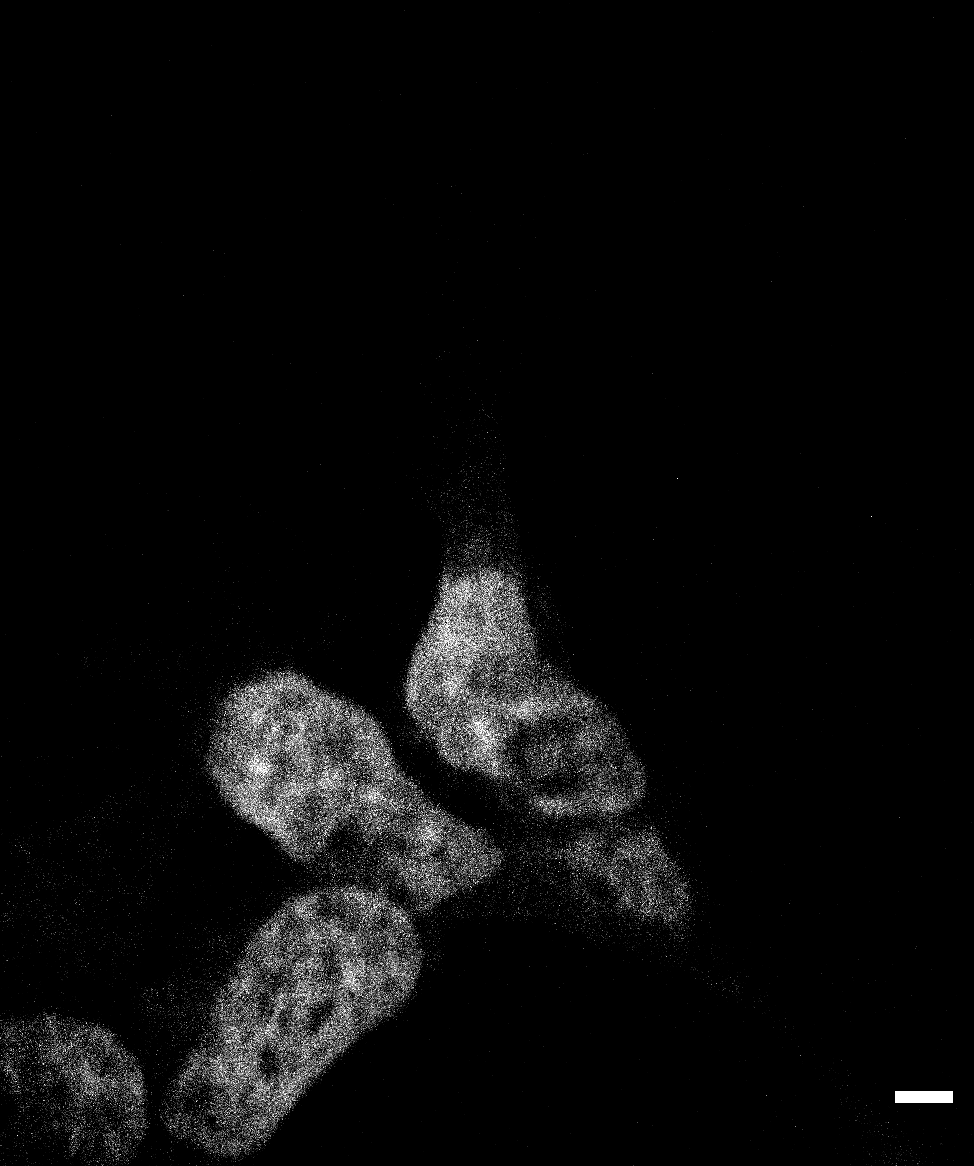

Supplement: Supplementary file 8 — Source data Fig. 2 [file 44320_2025_96_MOESM8_ESM.zip › SD_Images_Fig2K/MAX_4C9_DAPI.png]

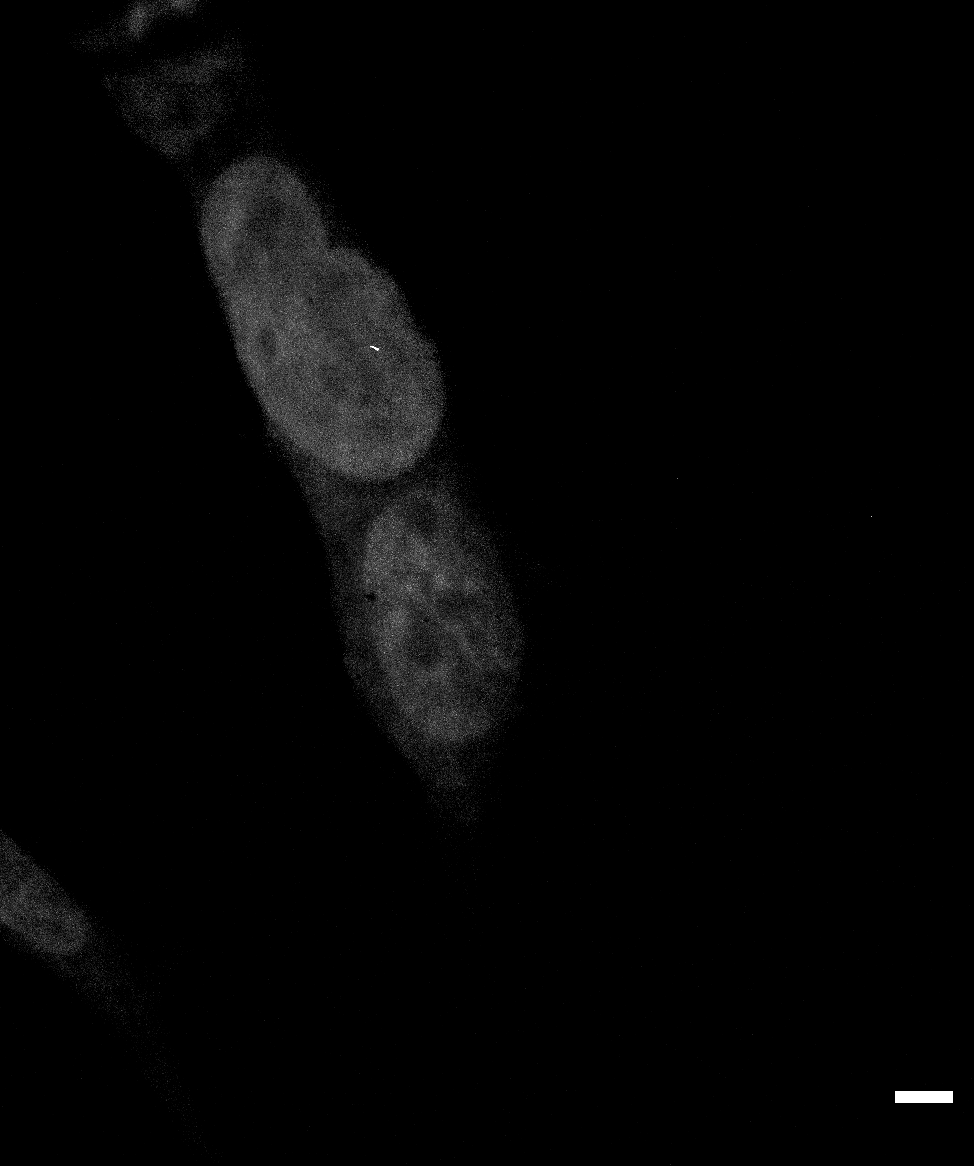

Supplement: Supplementary file 8 — Source data Fig. 2 [file 44320_2025_96_MOESM8_ESM.zip › SD_Images_Fig2K/MAX_3E10_DAPI.png]

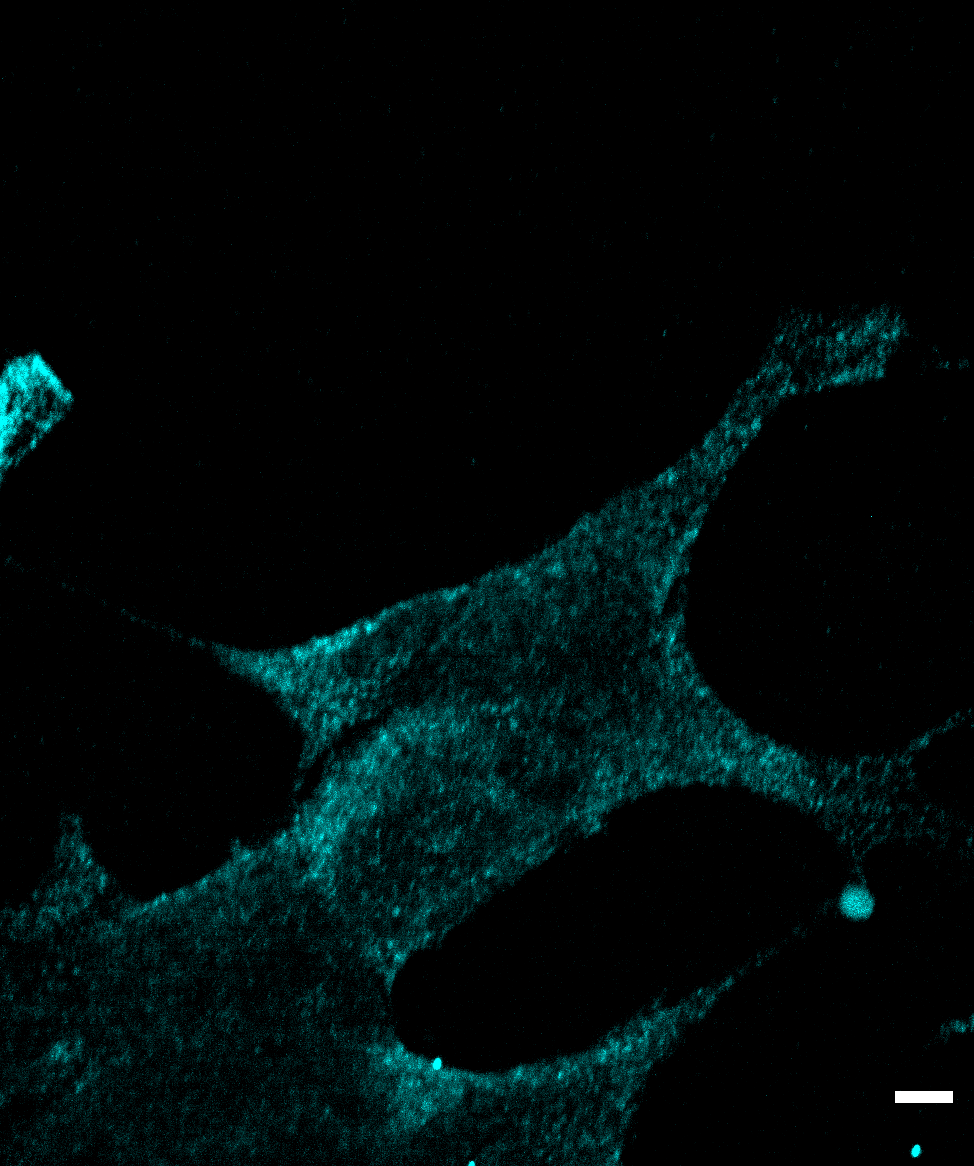

Supplement: Supplementary file 8 — Source data Fig. 2 [file 44320_2025_96_MOESM8_ESM.zip › SD_Images_Fig2K/MAX_2B7_HTT.png]
