# Supplementary material for: Multi-epitope immunocapture of huntingtin reveals striatum-selective molecular signatures
Source: Mol Syst Biol. 2025 Apr 1;21(5):492–522. doi: 10.1038/s44320-025-00096-3 (PMC12048488; doi:10.1038/s44320-025-00096-3)
Supplement: Supplementary file 9 — Expanded View Figures [file 44320_2025_96_MOESM9_ESM.pdf]

Expanded View Figures

Figure EV1. Evaluating IP efficiency and specificity of HTT antibodies for isolation of endogenous WT and mutant HTT from whole brain lysates of HD mouse models. ▶

(**A**) Domain map of HTT with IP antibodies marking their approximate epitope regions (see Materials and Methods). Blue cylinders represent the 7 HEAT repeat domains of HTT. Antibody IP efficiency was evaluated in Q20-8 weeks whole brain by western blotting using Odyssey Infrared detection (*bottom*). Antibodies in blue text had sufficient IP efficiency for further evaluation. FT = flow-through fraction, E = eluted fractions. (**B**) Western blot densitometry quantification of percent of HTT depleted from the flow-through (FT) vs. mIgG control lanes, and amount of HTT recovered from the elution (E) vs. mIgG control lanes. Antibodies are ordered (top to bottom) by epitope location (N-terminal to C-terminal). (**C**) Heatmap of the top 5% most abundant HTT interacting proteins ($n = 79$) isolated and quantified by IP-MS from whole brain using different HTT antibodies (EPR5526, 2B7-4C9, 4E10-3E10, 138-129). Raw MS protein abundances were normalized by their respective replicate medians and theoretical number of tryptic peptides and expressed as the $\log_2$ average of two biological replicates in Q20 and Q140-8 weeks conditions. Normalized protein abundances were analyzed by hierarchical clustering (distance metric: Euclidean and method: Ward) with no additional normalization or scaling. Missing values were imputed with the minimum quantified value. (**D**) Violin plot of $\log_2$ enrichment ratio (HTT IP vs mIgG IP) distribution for proteins co-isolated with HTT Q20 or Q140 from the striatum (8 weeks) using antibodies EPR5526, 2B7-4C9 pair, 4E10-3E10 pair, and D7F7. Enrichment ratio of HTT bait is indicated by orange circle. (**E**) Comparison of Htt, Hap40 (F8a1), and Synj1 levels in IPs of HTT Q20 from the striatum of 8 weeks old mice using EPR5526, 2B7-4C9, or 4E10-3E10 antibodies ($n = 4$, mean ± SD). Protein abundances were calculated as in (**C**). (**F**) Sequence alignment of Htt N17, corresponding to the EPR5526 epitope region, and Synj1 (aa. 399–408). The predicted crystal structure (AlphaFold 2.0) is shown, labeled with selected aligned amino acids of Synj1/HTT in an alpha-helix (dark blue), which forms hydrogen bond contacts with a proximal alpha-helix (light blue). (**G**) Scatterplot comparisons of co-isolated (prey) protein $\log_2$ enrichment ratios (HTT vs mIgG IP) in Q20 vs Q140 for N-terminal (left) and Central (right) antibody sets. Bait HTT and selected positive control interactions are denoted in red and orange circles, respectively. HTT interactions that were annotated in at least 2/3 of the previously reported HTT IP-MS studies from mouse brain are indicated in light blue circles. (**H**) Scatterplot of $\log_{10}$ abundance (MS summed intensity) for HTT vs. HAP40 showing high correlation in all individual IP-MS experiments by tissue. (**I**) Principal component analysis of all co-isolated $\log_2$ protein abundances stratified by antibody set (N-terminal and Central) and tissue. Abundances were median normalized. (**J**) Same as (**I**), except stratified by antibody set and polyQ length, for striatum (left), cortex (middle), and cerebellum (right).

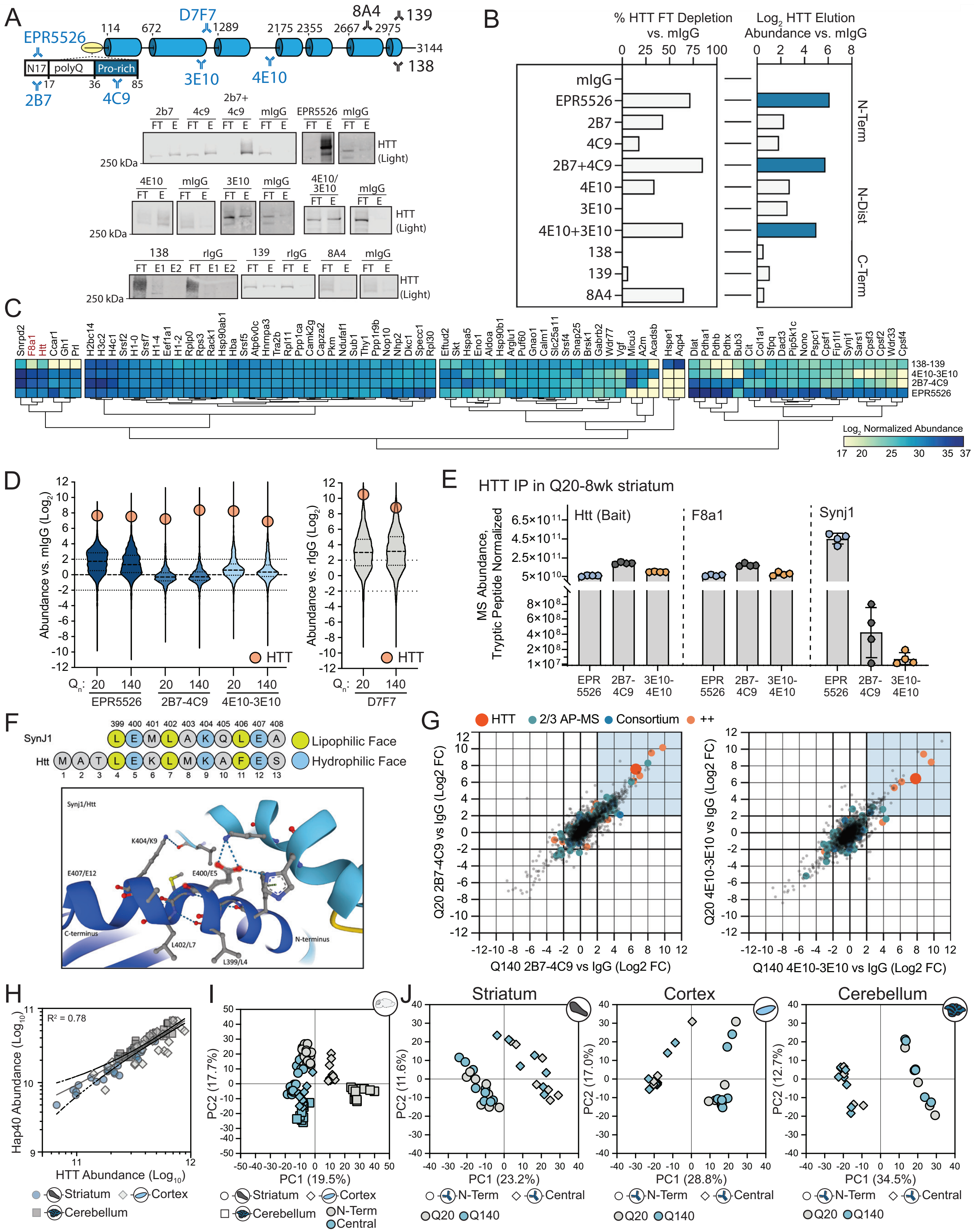
A
EPR5526
D7F7
8A4
139
138
114
672
1289
2175 2355
2667
2975
3144
N17
polyQ
Pro-rich
17
36
85
2B7
4C9
3E10
4E10
250 kDa
HTT (Light)
B
% HTT FT Depletion vs. mIgG
Log2 HTT Elution Abundance vs. mIgG
mIgG
EPR5526
2B7
4C9
2B7+4C9
4E10
3E10
4E10+3E10
138
139
8A4
N-Term
N-Dist
C-Term
C
138-139
4E10-3E10
2B7-4C9
EPR5526
Log2 Normalized Abundance
D
Abundance vs. mIgG (Log2)
Abundance vs. rIgG (Log2)
HTT
EPR5526
2B7-4C9
4E10-3E10
D7F7
E
HTT IP in Q20-8wk striatum
MS Abundance, Tryptic Peptide Normalized
Htt (Bait)
F8a1
Synj1
F
SynJ1
Htt
Lipophilic Face
Hydrophilic Face
Synj1/Htt
G
HTT
2/3 AP-MS
Consortium
++
Q20 2B7-4C9 vs IgG (Log2 FC)
Q140 2B7-4C9 vs IgG (Log2 FC)
Q20 4E10-3E10 vs IgG (Log2 FC)
Q140 4E10-3E10 vs IgG (Log2 FC)
H
R² = 0.78
Hap40 Abundance (Log10)
HTT Abundance (Log10)
Striatum
Cortex
Cerebellum
I
PC1 (19.5%)
PC2 (17.7%)
N-Term
Central
J
Striatum
Cortex
Cerebellum
PC1 (23.2%)
PC2 (11.6%)
PC1 (28.8%)
PC2 (17.0%)
PC1 (34.5%)
PC2 (12.7%)
Q20
Q140

 © The Author(s)

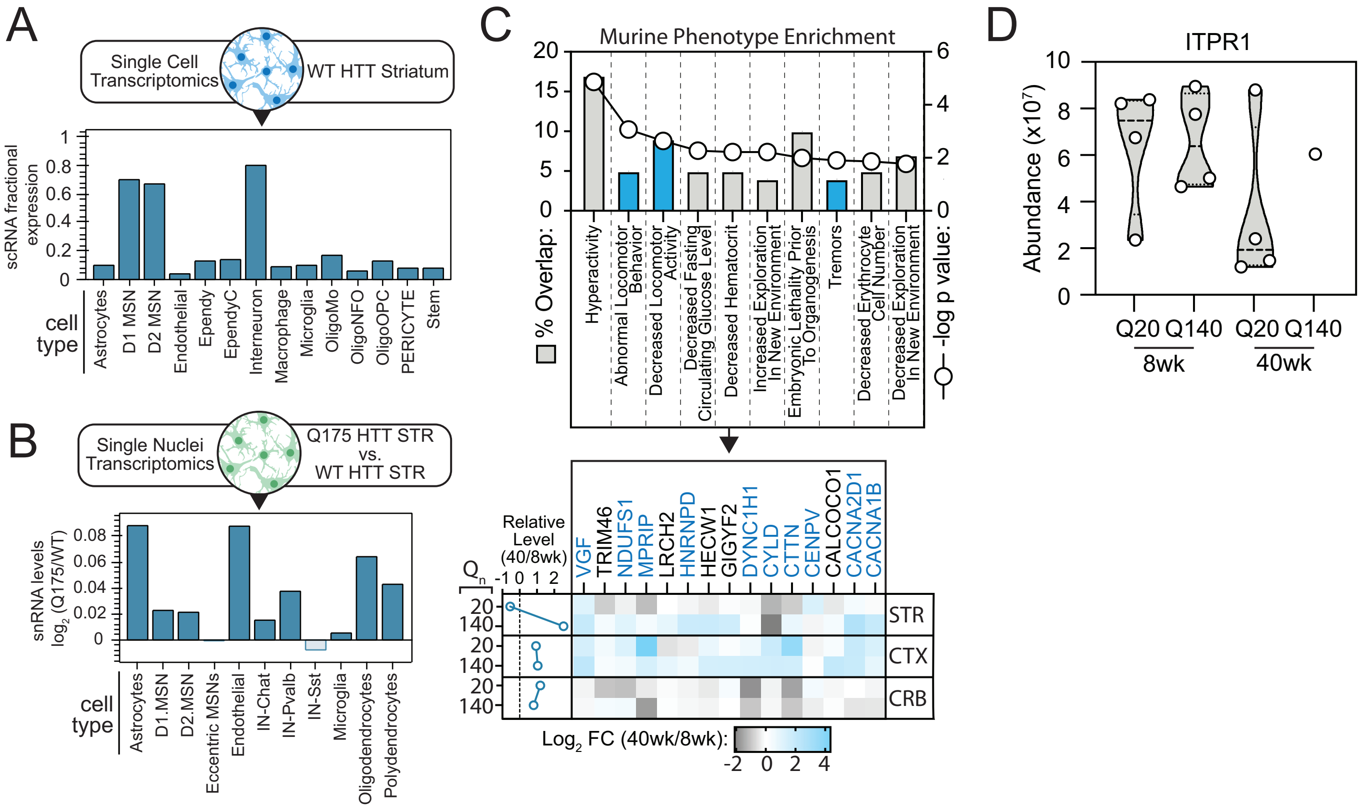

Figure EV2. Multiomics and functional ontology analysis of striatum-enriched HIP genetic modifiers.

(**A**, **B**) Preferential single cell type RNA fractional expression (**A**) and mHTT-induced single nuclei RNA levels (**B**) of proteins from STRING network in Fig. 7D. (**C**) Functional overrepresentation of murine phenotypes from proteins in Fig. 7D (top) and the corresponding annotated proteins with mHTT-dependent changes in interaction levels in the striatum, cortex, and cerebellum. Over-represented pathways were determined by EnrichR (https://maayanlab.cloud/Enrichr/) using a Fisher's Exact test and corrected for multiple hypothesis testing using the Benjamini-Hochberg method. (**D**) Interaction levels of ITRP1 with HTT determined by quantitative IP-MS. Individual replicates (n = 4) and median protein abundance values are shown.
